# The impact of preoperative nutritional status, intraoperative fluid administration volume, operating room temperature, and anesthesia duration on intraoperative hypothermia in elderly patients undergoing total joint replacement under general anesthesia: a logistic regression analysis and nursing intervention strategies

**DOI:** 10.3389/fmed.2025.1672165

**Published:** 2026-01-05

**Authors:** Mingming Lu, Tingting Liu, Jian Wang, Liu Wang, Hui Ni

**Affiliations:** 1 Department of Central Operating Room, Nanjing Tongren Hospital, School of Medicine, Southeast University, Nanjing, China; 2 Department of Anesthesiology, Nanjing Tongren Hospital, School of Medicine, Southeast University, Nanjing, China

**Keywords:** elderly, joint replacement surgery, general anesthesia, intraoperative hypothermia, nutritional status

## Abstract

**Objective:**

To analyze the factors associated with intraoperative hypothermia (IHC) in elderly patients undergoing total joint replacement under general anesthesia based on preoperative nutritional status, intraoperative fluid administration volume, operating room temperature, and anesthesia duration, and to construct and validate a risk prediction model.

**Methods:**

A retrospective study was conducted on 120 elderly patients who underwent joint replacement surgery at our hospital between March 2023 and March 2025. All patient data were obtained from the electronic medical record system, and patients were divided into an IHC group and a non-IHC group based on whether they experienced IHC. Patient-related data were collected and compared. Selection operator (LASSO) regression analysis and multivariate logistic analysis were performed to identify risk factors for IHC in elderly patients undergoing joint replacement surgery. A prediction model was established using R software and validated.

**Results:**

A total of 120 elderly patients who underwent joint replacement surgery at our hospital were included in this study. According to medical record system records, 21 patients developed IHC, accounting for 17.50%, and were included in the IHC group. Ninety-nine patients did not develop IHC, accounting for 82.50%, and were included in the non-IHC group. There were significant statistical differences between the IHC group and the non-IHC group in terms of BMI, intraoperative fluid administration volume, operating room temperature, anesthesia duration, serum albumin (ALB), and total protein (TP; *p* < 0.05). After LASSO regression analysis, none of the above indicators exhibited multicollinearity or overfitting. Logistic regression analysis revealed that intraoperative fluid administration volume and anesthesia duration were risk factors for IHC in elderly joint replacement surgery patients (OR = 1.002, 142.629, *p* < 0.05), Operating room temperature and ALB were protective factors for IHC in elderly joint replacement surgery patients (OR = 0.626, 0.712, *p* < 0.05); Based on the results of the logistic regression analysis, a risk prediction model for IHC in elderly joint replacement surgery patients was constructed using a nomogram. The ROC curve showed an AUC value of 0.994 (95% CI: 0.985–1.000). While this indicates excellent discriminative ability in the current cohort, we acknowledge that such a high value may reflect overfitting due to the limited sample size. External validation is required to confirm the model’s generalizability. The calibration curve indicated that the model’s predicted results were well aligned with the actual occurrence of IHC in patients. The Cox-Snell R^2^ was 0.89, Nagelkerke R^2^ was 0.538, Brier Score was 0.027, model fit *p*-value was 1.00, and the statistic was 0.08. The clinical decision curve was generally above the two extreme curves, indicating that the factors included in the nomogram have a high net benefit in predicting IHC occurrence in patients.

**Conclusion:**

There are numerous factors associated with the occurrence of IHC in elderly patients undergoing joint replacement surgery, primarily including intraoperative fluid administration volume, operating room temperature, anesthesia duration, and ALB. The risk prediction nomogram model constructed based on these factors demonstrates certain predictive value for the risk of IHC occurrence. Clinically, early screening of high-risk populations and implementation of targeted nursing interventions hold significant implications for clinical outcomes.

## 1 Introduction

With the acceleration of population aging and advancements in medical technology, the number of elderly patients undergoing joint replacement surgery has shown a significant upward trend (1, 2). Previous studies have indicated that joint replacement surgery is a well-established treatment method that involves surgically replacing severely diseased or damaged joints with artificial joint prostheses to restore joint function and alleviate pain. It is primarily indicated for patients with severe joint dysfunction caused by conditions such as osteoarthritis, rheumatoid arthritis, and avascular necrosis of the femoral head (3, 4). Vandepitte et al. (5) noted that joint replacement surgery can effectively alleviate pain, restore joint function, and significantly improve the quality of life for elderly patients. However, due to their unique physiological characteristics, elderly patients face a high risk of intraoperative hypothermia (IHC), a common and serious complication, during the prolonged and highly invasive joint replacement surgery process. Studies have shown that the incidence of IHC in elderly patients undergoing joint replacement surgery under general anesthesia can exceed 50%, far higher than in other age groups or patients undergoing minor surgeries. Even seemingly minor temperature changes pose multifaceted and profound threats to the physiological homeostasis and postoperative recovery of elderly surgical patients. For this study, elderly was defined as patients aged 65 years or older, consistent with the World Health Organization’s classification. This population exhibits age-related physiological changes that impair thermoregulation, including reduced metabolic rate, diminished vasoconstriction response, and decreased subcutaneous fat insulation (6). Patients with comorbidities such as diabetes may experience further impairment in autonomic thermoregulation due to peripheral neuropathy and microvascular dysfunction.

Previous studies have indicated that IHC has widespread and profound pathophysiological effects, including inducing chills, significantly increasing oxygen consumption and carbon dioxide production, and exacerbating cardiovascular and pulmonary burden; simultaneously inhibiting platelet function and the activity of multiple enzymes involved in the coagulation cascade, significantly prolonging coagulation time, increasing bleeding at surgical incisions and perioperative blood loss, and even increasing the risk of myocardial ischemia, posing particular danger to elderly patients with concomitant cardiovascular diseases (7, 8). Furthermore, according to relevant literature, IHC impairs neutrophil function, reduces skin blood flow and oxygenation, slows liver metabolism and renal excretion, weakens the body’s anti-infective defense capabilities, and prolongs the duration of action of anesthetic drugs and muscle relaxants, significantly increasing the risks of surgical site infections, pulmonary infections, and delayed awakening, leading to prolonged hospital stays, impaired recovery, and increased medical costs (9). Additionally, IHC can trigger a stress response characterized by activation of the sympathetic nervous system. This sympathetic activation increases myocardial oxygen demand and can induce vasoconstriction, thereby elevating the risk of myocardial ischemia, particularly in elderly patients with pre-existing cardiovascular compromise (7, 8). The cold-induced diuresis associated with IHC may further contribute to hemodynamic instability by reducing intravascular volume. Therefore, effective prevention and management of IHC are critical components in ensuring perioperative safety and optimizing surgical outcomes for elderly joint replacement patients. Fu et al. (10) suggested that nutritional indicators such as serum albumin (ALB) and total protein (TP) levels may serve as important biomarkers for predicting thermal regulation capacity. Additionally, the direct infusion of unwarmed room-temperature or refrigerated fluids into the circulatory system is one of the primary sources of “cold load” leading to rapid and direct decreases in core body temperature. Additionally, based on relevant research findings, operating rooms are typically maintained at lower temperatures to meet sterile operating conditions and ensure the comfort of surgical staff, but this significantly increases heat loss through radiation and convection, which are key drivers of surface heat loss (11, 12). Anesthetic drugs themselves suppress the hypothalamic temperature regulation center, widening the temperature regulation threshold range, while also prolonging the duration of surface exposure and heat loss (13, 14). Although previous studies have identified various potential factors associated with IHC, and routine warming measures have been incorporated into clinical practice guidelines, there remains a lack of advanced statistical methods to quantitatively assess the independent contributions, interactions, and combined predictive efficacy of these four key factors in elderly joint replacement patients.

Based on this, this study deepens our understanding of the mechanisms of IHC in elderly surgical patients, particularly by quantifying the impact of key controllable factors. It provides new perspectives and methods for perioperative temperature management research and emphasizes the core value of nursing in perioperative medicine, contributing to improving the overall care quality and outcomes for elderly joint replacement surgery patients.

## 2 Materials and methods

### 2.1 Declaration of ethical standards

This study was approved by the review committee and ethics committee of this institution. Given that this study is a retrospective study and only uses de-identified research subject data, informed consent is not required as there is no risk or adverse effect on the research subjects. This exemption complies with regulations and ethical guidelines related to retrospective studies.

### 2.2 Study design

**Figure 1:**
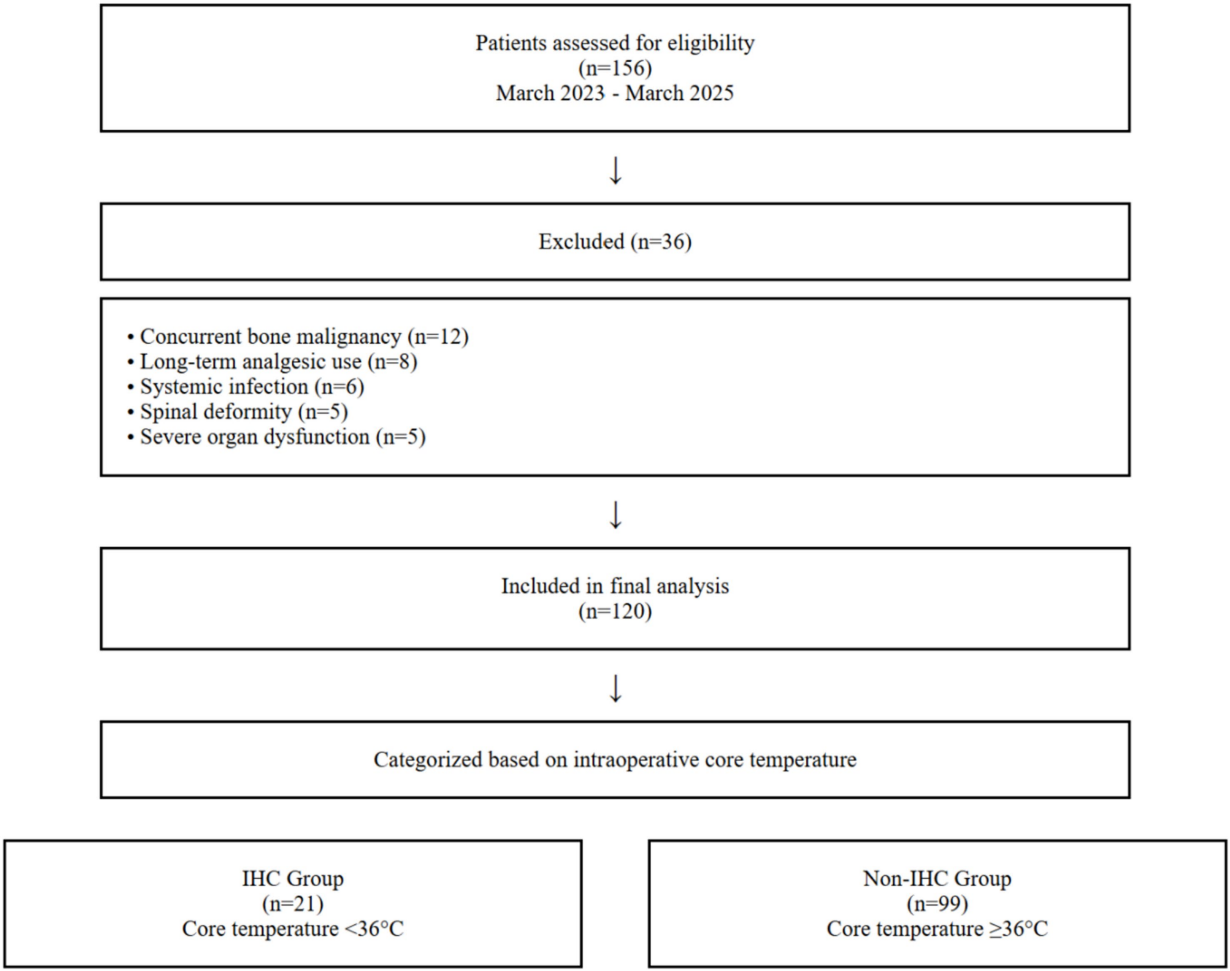
Patient flow diagram. It illustrates the screening, inclusion, and exclusion process of study participants. The diagram details the number of patients assessed for eligibility, excluded with specific reasons, and ultimately included in the final analysis for the IHC and non-IHC groups.

This retrospective study included 120 elderly patients who underwent joint replacement surgery at our hospital between March 2023 and March 2025. All patient data were obtained from the electronic medical record system and were categorized into an IHC group and a non-IHC group based on the occurrence of IHC. A patient flow diagram was constructed according to the STROBE guidelines for observational studies (Figure 1). During the study period from March 2023 to March 2025, a total of 156 elderly patients undergoing joint replacement surgery were initially assessed for eligibility. After applying the inclusion and exclusion criteria, 36 patients were excluded (12 due to concurrent bone malignancy, 8 due to long-term analgesic use, 6 due to systemic infection, 5 due to spinal deformity, and 5 due to severe organ dysfunction). The final analysis included 120 patients who met all criteria and were categorized into IHC (*n* = 21) and non-IHC (*n* = 99) groups based on intraoperative core temperature measurements.

This retrospective study included 120 elderly patients who underwent primary unilateral total joint replacement surgery at our hospital between March 2023 and March 2025. The procedures included total knee arthroplasty (TKA, *n* = 68), total hip arthroplasty (THA, *n* = 42), and total shoulder arthroplasty (TSA, *n* = 10). All patients received general anesthesia according to standardized institutional protocols. All included cases were primary joint arthroplasties performed for degenerative joint diseases. Complex primary cases requiring specialized implants or techniques were excluded to maintain homogeneity. Revision arthroplasties were also excluded due to their typically longer operative times and different surgical characteristics that could confound hypothermia risk assessment.

### 2.3 Inclusion and exclusion criteria

Inclusion criteria: (1) Age ≥ 65 years, consistent with the World Health Organization definition of elderly population; (2) Diagnosed with end-stage osteoarthritis or rheumatoid arthritis requiring joint replacement; (3) Scheduled for primary unilateral total joint arthroplasty under general anesthesia; (4) Complete preoperative and intraoperative medical records available; (5) American Society of Anesthesiologists (ASA) physical status I-III.

Exclusion criteria: (1) Age < 65 years; (2) Revision joint arthroplasty or bilateral procedures; (3) Regional anesthesia (spinal or epidural) instead of general anesthesia; (4) Pre-existing thermoregulatory disorders or thyroid dysfunction; (5) Active systemic infection or inflammatory conditions; (6) Severe hepatic or renal impairment (Child-Pugh class C or eGFR < 30 mL/min/1.73m^2^); (7) Cognitive impairment preventing informed consent or cooperation; (8) Emergency surgery or traumatic fractures.

### 2.4 Classification criteria

Nasal pharyngeal temperature measurement is most commonly used in patients undergoing general anesthesia with endotracheal intubation and can also be applied to patients receiving laryngeal mask ventilation. This is because the measurement site is closer to the brain, accurately reflecting core temperature, and is easier to perform than esophageal temperature measurement. During surgery, the temperature-sensitive probe of the monitor is placed in the patient’s nasopharynx to measure core body temperature from the start of anesthesia until the end of surgery. The measurement depth is the distance from the nasal wing to the same-side mandibular angle, and the temperature-sensitive probe is securely fixed with adhesive tape. From the start of anesthesia until the end of surgery, the nasopharyngeal temperature is recorded every 5 min, with a core body temperature <36 °C serving as the diagnostic criterion for intraoperative hypothermia.

### 2.5 General data collection

Data on study subjects were retrospectively collected through the electronic medical record system, primarily including gender (male/female), age, body mass index (BMI), hypertension [meeting the diagnostic criteria in The Japanese Society of Hypertension Guidelines for Self-monitoring of Blood Pressure at Home (Second Edition) (15)] (present/absent), diabetes [meeting the diagnostic criteria in the “Application of the Chinese Expert Consensus on Diabetes Classification in Clinical Practice” (16)] (yes/no), hyperlipidemia [meeting the diagnostic criteria in the “Report of the Japan Atherosclerosis Society (JAS) Guideline for Diagnosis and Treatment of Hyperlipidemia in Japanese Adults” (17)] (yes/no), affected side (left/right), preoperative heart rate, preoperative diastolic blood pressure, preoperative systolic blood pressure, body temperature of patients upon admission to the operating room, intraoperative fluid administration volume, operating room temperature, and anesthesia duration.

### 2.6 Laboratory indicators

Collect 5 mL of fasting venous blood from the patient in the morning. Use a centrifuge (model: LL900, manufacturer: Luoyang Hongshi Machinery Equipment Co., Ltd.) at 3000 rpm for 10 min to separate the patient’s serum. Using the immunoturbidimetric method, measure the patient’s fasting plasma glucose (FPG) using a fully automatic biochemical analyzer (Model: BS-850, Manufacturer: Mindray Medical International Co., Ltd.). Use a fully automatic blood biochemical analyzer (Model: Boke BK-200, Manufacturer: Shandong Boke Bio-Industry Co., Ltd.) to measure the patient’s albumin (ALB) and total protein (TP). In this study, ALB and TP are used as the primary laboratory indicators for assessing the patient’s preoperative nutritional status.

### 2.7 Statistical analysis

This study used SPSS 26.0 and R 4.3.2 software for statistical analysis and graphical representation. For continuous variables that followed a normal distribution, an independent samples t-test was used, with results expressed as (x̅ ± s); for those that did not, a Mann–Whitney U test was used, with results expressed as median M (P25, P75). Categorical data were analyzed using the chi-square (χ^2^) test and expressed as frequencies. A *p*-value < 0.05 was considered statistically significant. To reduce the impact of multicollinearity on regression results and avoid overfitting, the selected factors were analyzed using the “glmnet” package in R software based on the least absolute and selected operator (Lasso) regression analysis. The final selected variables were included in a multivariate logistic regression analysis to identify independent risk factors for IHC occurrence in patients. The “rms” package in R was used to construct a regression model for prediction. The model was validated using the Bootstrap method with 1,000 repeated samples and validation set data. The area under the receiver operating characteristic (ROC) curve (AUC) was used to assess the model’s discriminatory ability; a standard calibration curve was plotted and the Brier score was calculated to assess the model’s calibration; and decision curve analysis (DCA) was conducted to evaluate the model’s clinical net benefit.

## 3 Results

### 3.1 Comparison of general data between the IHC group and the non-IHC group

A total of 120 elderly patients who underwent joint replacement surgery at our hospital were included in this study. According to the medical record system, 21 patients developed IHC, accounting for 17.50%, and were included in the IHC group, while 99 patients did not develop IHC, accounting for 82.50%, and were included in the non-IHC group. There were significant statistical differences between the IHC group and the non-IHC group in terms of BMI, intraoperative fluid administration volume, operating room temperature, anesthesia duration, ALB, and TP (*p* < 0.05). However, there were no significant statistical differences between the two groups in terms of age, gender, and other variables (*p* > 0.05), as shown in Table 1.

**Table 1:** Comparison of general data of patients in the IHC group and the non-IHC group.

Indicator		IHC group (*n* = 21)	No IHC group (*n* = 99)	*x^2^/t*	*p*
Gender (n)	Male	7	24	0.747	0.387
	Female	14	75		
Age (years)		81.05 ± 13.89	73.38 ± 18.77	1.770	0.079
BMI (kg/m^2^)		21.90 ± 1.29	24.54 ± 2.89	4.090	<0.001
Hypertension (n)	Have	16	59	2.036	0.154
	No	5	40		
Diabetes (n)	Have	4	9	1.778	0.182
	No	17	90		
Hyperlipidemia (n)	Have	0	1	0.214	0.644
	No	21	98		
Affected side (n)	On the left	10	49	0.024	0.876
	Right side	11	50		
Preoperative heart rate (beats/min)		79.67 ± 8.33	77.39 ± 8.41	1.130	0.261
Preoperative diastolic blood pressure (mmHg)		80.29 ± 8.11	82.79 ± 8.34	1.253	0.213
Preoperative systolic blood pressure (mmHg)		155.24 ± 9.01	155.08 ± 8.42	0.078	0.938
Body temperature of patients entering the room (°C)		36.45 ± 4.19	36.49 ± 4.11	0.040	0.968
Intraoperative infusion volume (mL)		1451.77 ± 166.35	1257.68 ± 132.47	5.821	<0.001
Operating room temperature (°C)		20.84 ± 2.20	23.78 ± 2.41	5.151	<0.001
Anesthesia time (h)		2.82 ± 0.33	2.35 ± 0.28	6.767	<0.001
FPG (mmol/L)		6.45 ± 1.06	6.97 ± 1.21	1.825	0.071
ALB (g/L)		28.35 ± 5.12	39.17 ± 8.88	5.385	<0.001
TP (g/L)		57.09 ± 5.92	63.99 ± 6.74	4.346	<0.001

### 3.2 Analysis of important variables associated with IHC in elderly patients undergoing joint replacement surgery

**Figure 2:**
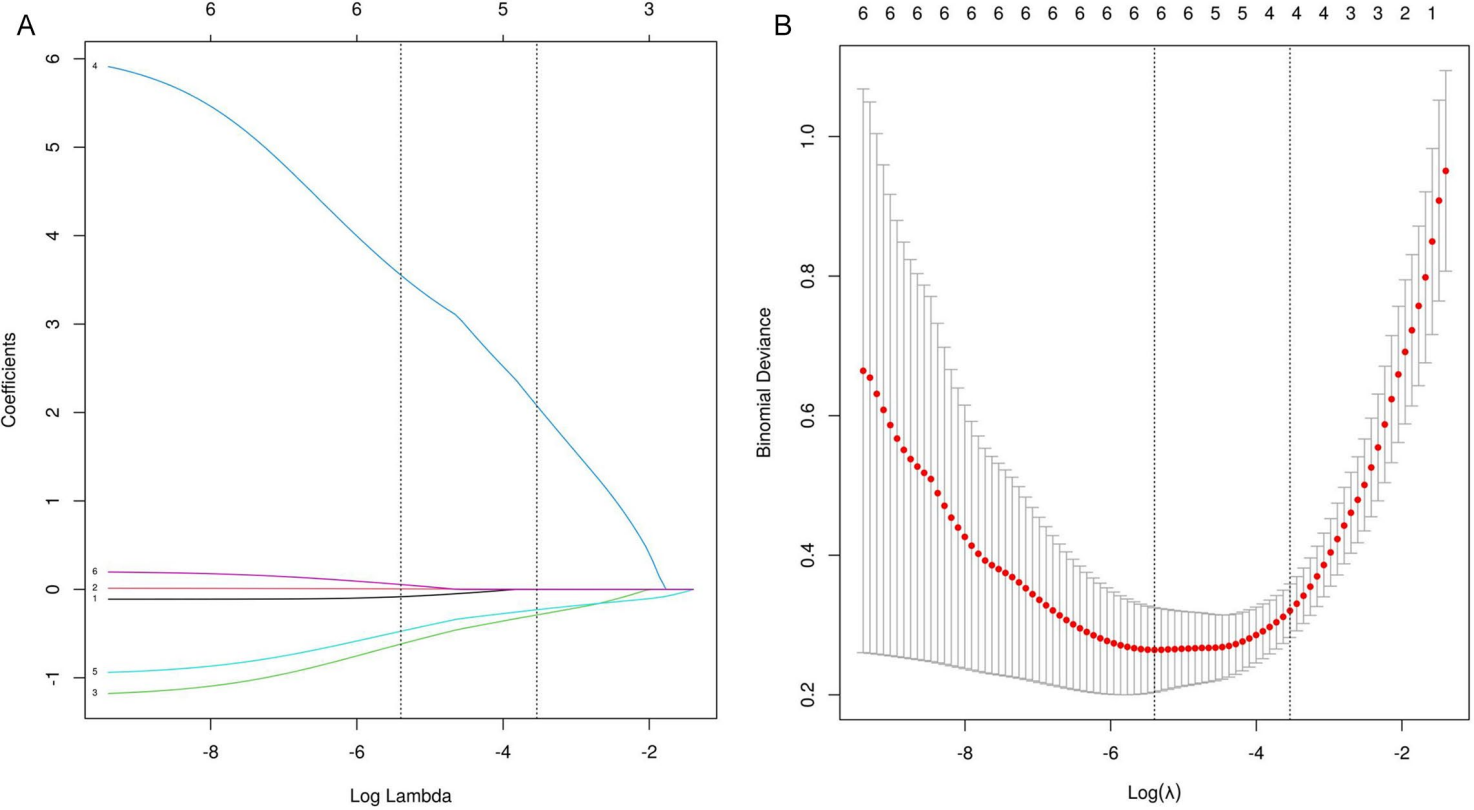
**(A,B)** LASSO regression analysis. **(A)** Uses a dual horizontal axis design, with the lower axis displaying the logarithmic value of log(*λ*) and the upper axis displaying the number of retained variables. The vertical axis presents the standardized coefficients of each feature, with their change trajectories intuitively displayed through colored curves. **(B)** Shows the mean squared error on the vertical axis. The red vertical line indicates the optimal variable subset corresponding to the minimum error value λ.min, which represents the number of independent variables in the model when the mean squared error is minimized. The black vertical line λ.lse represents the simplified model selection when the error increases by one standard error.

To optimize model performance and address multicollinearity issues, the study employed LASSO regression for feature selection of the initially screened differential variables (Figure 2A), compressing the regression coefficients of redundant variables to 0. To validate the reliability of the LASSOlogit results, cvlassologit cross-validation was used to determine the optimal *λ* (Figure 2B). The best fitting results were obtained when λ was 0.005 for minimum mean square error and 0.029 for minimum standard error of distance. Four variables were included: intraoperative fluid administration volume, operating room temperature, anesthesia duration, and ALB.

### 3.3 Logistic regression analysis of IHC in elderly patients undergoing joint replacement surgery

The variables selected by LASSO regression were included in the logistic analysis. The results indicated that intraoperative fluid administration volume and anesthesia duration were both risk factors for IHC in elderly patients undergoing joint replacement surgery (OR = 1.002, 142.629, *p* < 0.05). While operating room temperature and albumin (ALB) were protective factors for IHC in elderly joint replacement surgery patients (OR = 0.626, 0.712, *p* < 0.05) (See Table 2).

**Table 2:** Logistic regression analysis affecting IHC in elderly patients undergoing joint replacement surgery.

Indicator	*β* value	SE	Wald χ^2^	*p* value	OR value	95% CI for OR
Intraoperative infusion volume	0.002	0.001	6.256	0.012	1.002	1.000–1.003
Operating room temperature	−0.468	0.142	10.868	0.001	0.626	0.474–0.827
Anesthesia time	4.960	1.199	17.112	<0.001	142.629	13.600–1495.831
ALB	−0.339	0.066	26.553	<0.001	0.712	0.626–0.810

### 3.4 Establishment of a risk prediction model for IHC in elderly patients undergoing joint replacement surgery

**Figure 3:**
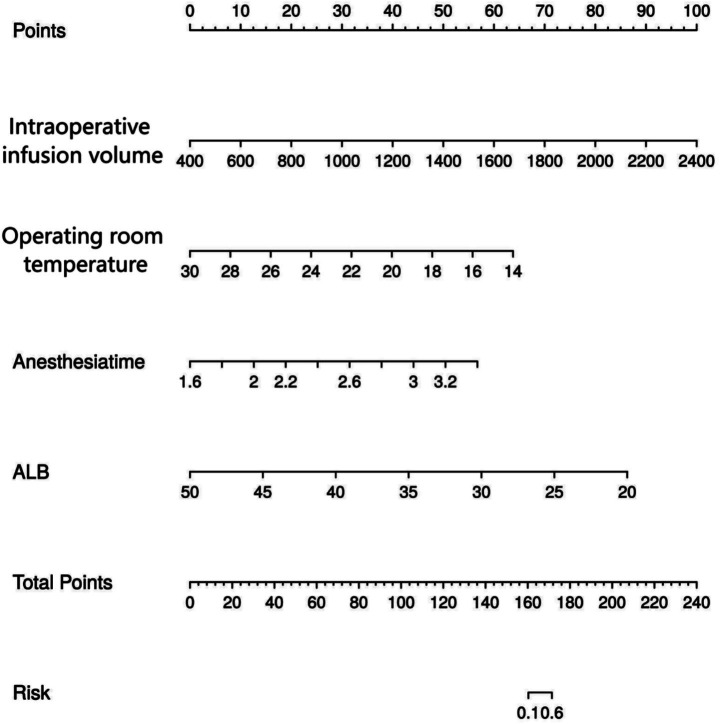
Risk prediction nomogram of IHC in elderly patients undergoing joint replacement surgery.

Based on the results of the statistical analysis, the following regression model was established: Logit(P) = 5.434 + 0.002 × intraoperative fluid administration volume –0.468 × operating room temperature + 4.960 × anesthesia duration –0.339 × ALB. Based on the four independent predictive factors identified through screening, a visual scoring system was developed. Each risk factor corresponds to an independent scale axis, with the scale length intuitively reflecting the weight of that factor’s contribution to the prognosis. The total score is obtained by summing the scores corresponding to each variable, and the individualized risk prediction value is mapped onto the probability scale, enabling a quantitative assessment of IHC risk prediction in elderly joint replacement surgery patients, as shown in Figure 3.

### 3.5 Model performance evaluation

#### 3.5.1 ROC curve

**Figure 4:**
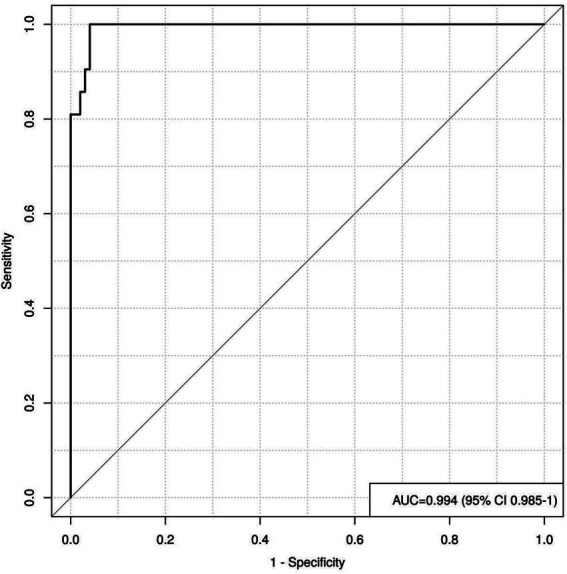
Shows the area under the ROC curve of the nomogram model.

By plotting the ROC curve, we can see that the AUC value is 0.994, with a 95% CI of 0.985 to 1.000, indicating that the reclassified curve model has good discriminatory power, as shown in Figure 4.

#### 3.5.2 Calibration curve and decision curve

**Figure 5:**
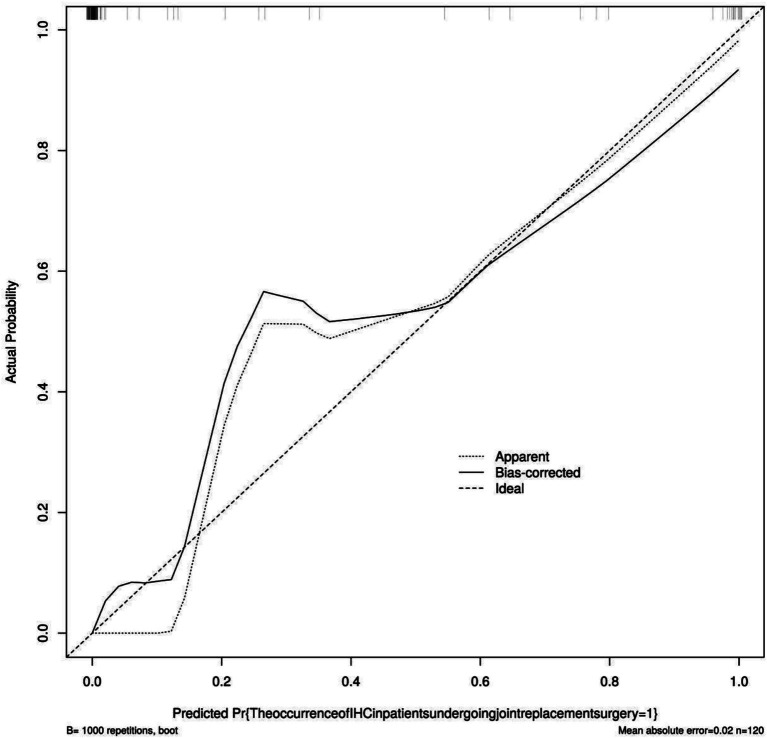
Calibration curve of the nomogram model.

**Figure 6:**
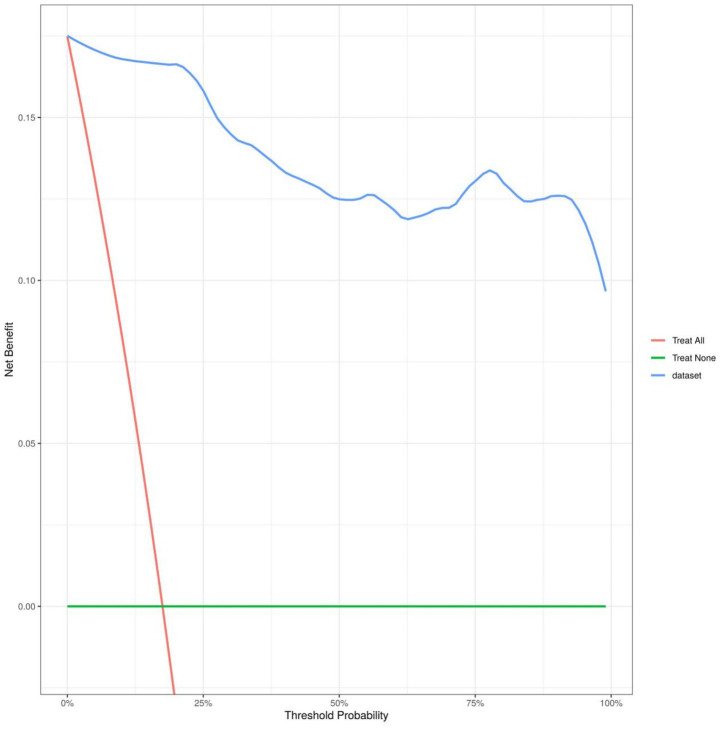
The decision curve of the nomogram model.

The model was validated using the Bootstrap method with 1,000 repeated samples. The Cox-Snell R^2^ was 0.89, the Nagelkerke R^2^ was 0.538, the Brier Score was 0.027, and the model fit *p*-value was 1.00. The statistic is 0.08, indicating that the column chart model exhibits good calibration, as shown in Figure 5; the DCA curve is higher than the two extreme curves, indicating that the predicted net benefit of the relevant factors in the column chart is higher, as shown in Figure 6.

## 4 Discussion

IHC, defined as a core body temperature below 36 °C, is a frequent and clinically significant complication in elderly patients undergoing major surgery such as joint replacement. In our cohort, the incidence of IHC was 17.50%, which is lower than previously reported rates in similar populations (e.g., 39.05–56.83%) (18–20). This discrepancy may be attributed to differences in patient characteristics, surgical protocols, or warming measures. IHC is not merely a thermal disturbance; it has profound physiological consequences, including increased oxygen consumption, impaired coagulation, and elevated risks of surgical site infection and cardiovascular events (7–9, 21). Therefore, identifying modifiable risk factors and developing predictive models are critical for optimizing perioperative management. Our findings underscore the importance of maintaining adequate operating room temperature, a factor sometimes overlooked in clinical practice, as clearly demonstrated by its significant association with IHC risk in our cohort.

Our analysis identified four independent predictors of IHC: intraoperative fluid volume, operating room temperature, anesthesia duration, and serum ALB. These factors align with existing physiological knowledge. For instance, unwarmed intravenous fluids act as a direct cold stressor, while low ambient temperature and prolonged anesthesia exacerbate heat loss (22–24). Similarly, hypoalbuminemia reflects poor nutritional status and reduced thermoregulatory capacity (25–27). The strong association between ALB and IHC underscores the importance of preoperative nutritional assessment and intervention in high-risk patients.

According to the data from this study, there were significant statistical differences (*p* < 0.05) between the IHC group and the non-IHC group in terms of BMI, intraoperative fluid administration volume, operating room temperature, anesthesia duration, ALB, and TP. Logistic regression model analysis revealed that intraoperative fluid administration volume, anesthesia duration, operating room temperature, and ALB were all risk factors for IHC in elderly joint replacement surgery patients (*p* < 0.05). Intraoperative fluid medications are typically stored in a low-temperature convection environment, and medications used during surgery are not preheated. For every 2 liters of fluid administered that is below the ambient temperature, the body’s core temperature decreases by 1 °C, exhibiting a “cold dilution” effect. During surgery, large-volume fluid infusion increases the body’s heat loss, leading to a decrease in core body temperature and symptoms such as chills and shivering, significantly increasing the risk of IHC. Previous studies have shown that large-volume fluid infusion during surgery absorbs the patient’s body heat to maintain normal body temperature, leading to an increasing rate of heat loss during the infusion process, ultimately causing a decrease in body temperature and triggering IHC (22–24). Under normal circumstances, when core body temperature decreases by 3 °C, metabolic rate and oxygen consumption can decrease by 30–50%. The brain and liver are particularly sensitive to hypothermia, which can directly impair metabolic function in brain, liver, and cardiac tissues and cells, leading to ischemia, hypoxia, and reduced respiratory rate, heart rate, and mean arterial pressure. Carella et al. (28) reported that 1 h after general anesthesia, the body’s threshold for cold response decreases. Anesthetic drugs circulate through the bloodstream to the hypothalamus, inducing an anesthetic state that inhibits the body’s temperature regulation center, affecting heat production. This also reduces metabolic rate, suppresses neural reflexes, and impairs metabolic and circulatory functions, preventing the body from adjusting its temperature according to environmental changes. This disrupts temperature regulation, causing free heat to shift from the core to the periphery and lowering core body temperature. Therefore, the longer the duration of anesthesia, the higher the risk of IHC. Prolonged exposure to anesthetic agents not only disrupts central thermoregulation but also perpetuates peripheral vasodilation and heat loss to the environment. Previous studies have indicated that under normal conditions, the operating room humidity should be maintained between 55 and 60%, with a temperature of 24 °C to 26 °C (29, 30). When the operating room temperature is below 24 °C, it significantly reduces the hypothalamus’s ability to regulate temperature. Additionally, during surgery, a large area of skin is exposed to the environment, and when the operating room temperature is low, the temperature difference between the environment and the skin is significant, leading to rapid convective heat loss in the operating room air, causing the patient’s body temperature to continue to drop, increasing surface heat loss and the incidence of IHC. This highlights the critical role of maintaining recommended operating room temperatures (24 °C to 26 °C) as a simple yet effective preventive strategy against IHC. Previous studies have indicated that ALB is synthesized by the liver and is the primary protein component of total serum protein in healthy individuals. It maintains blood colloid osmotic pressure and facilitates the transport of metabolic substances within the body, serving as a common indicator of nutritional status (25, 26). Elevated preoperative ALB levels indicate greater fat reserves in the body, providing an additional insulating barrier and better thermal insulation; conversely, low levels indicate significantly reduced fat tissue content, faster heat loss, and heightened sensitivity to cold stimuli during surgery, making patients more prone to IHC during the procedure. Qian R et al. (27) suggested that a progressive decrease in preoperative ALB levels indicates reduced protein reserves, decreased heat production, and weakened immune defense, with relatively low fat tissue content in the body. This leads to a significant decline in the body’s ability to regulate heat loss, resulting in easier heat loss during surgery and a higher risk of IHC, which aligns with the findings of this study.

In this study, a regression model was constructed by combining various factors to create a regression line diagram, transforming complex multi-factor regression equations into graphical representations, thereby making abstract data outcomes visualizable and readable. Different predictive variables were used to assess the approximate probability of patients developing IHC. The AUC value of the ROC curve is 0.994, indicating that the model has excellent discriminative ability, accurately distinguishing between groups with and without IHC. The 95% CI (0.985–1.000) further confirms the reliability of this result. The calibration curve shows that the model’s predictive results align well with the actual occurrence of IHC, This means that the consistency between the model’s predictive results and the actual observed outcomes is high, further enhancing the model’s credibility. The clinical decision curve is generally higher than the two extreme curves, indicating that the factors included in the decision curve have a high net benefit in predicting the occurrence of IHC, providing stronger support for clinical decision-making. Given the risks associated with IHC occurrence and its related risk factors, the following clinical interventions can be implemented: ① Conduct a comprehensive preoperative assessment of the patient’s condition, develop an appropriate surgical plan, and ensure close collaboration between operating room staff and surgeons to minimize surgical duration, reduce anesthesia drug administration time, and maximize control of intraoperative bleeding while preventing hypothermia; ② Assess the patient’s nutritional status preoperatively and administer individualized preoperative treatment such as medical-grade multivitamin carbohydrates to improve nutritional status; ③ Preheat the fluids to be administered intraoperatively in a temperature-controlled incubator to 37 °C to prevent heat loss; ④ Turn on the operating room air conditioning 30 min before surgery to maintain an indoor temperature between 24 °C and 26 °C, and use a water-heated blanket to warm the operating table, maintaining a temperature of approximately 38 °C until the end of surgery; ⑤ Continuously monitor core body temperature (such as nasopharyngeal temperature, esophageal temperature, or bladder temperature) during surgery. If body temperature drops, promptly notify the physician and strengthen warming measures to avoid complications such as shivering or hypothermia; ⑥ During surgery, use high-insulation blankets or surgical drapes to cover non-surgical areas, avoid exposing non-surgical areas, and ensure that surgical drapes and bed linens in the surgical area remain dry to prevent moisture, reduce heat loss from the skin surface, and minimize the temperature difference between the environment and the skin to prevent hypothermia during surgery; ⑦ When disinfecting joint cavities during surgery, use 37 °C saline solution and apply hemostatic gauze soaked in warm saline solution for hemostasis. Simultaneously connect the patient’s airway tube to a humidifier to maintain constant humidity and temperature in the respiratory tract; ⑧ Apply surgical skin protection film to the surgical area to minimize heat loss.

Several practical factors specific to joint replacement surgery merit discussion regarding intraoperative temperature management. The choice between cemented and uncemented fixation presents a particular challenge. Cemented procedures often require lower ambient operating room temperatures (20–22 °C) to optimize cement viscosity and working time, potentially increasing patient heat loss. In our institution, cemented components were used in 85% of cases, primarily in older patients with osteoporotic bone. Additionally, the use of laminar airflow systems, while essential for infection prevention, creates convective heat loss that compounds temperature decline. All patients in this study received forced-air warming systems (Bair Hugger™) positioned beneath sterile drapes, regulated by anesthesia staff to maintain normothermia. However, the efficacy of these systems can be compromised by extensive surgical exposure and fluid administration. From an anesthesia perspective, the choice and depth of anesthetic agents, management of neuromuscular blockade, and strategies for hemodynamic stability all interact with thermoregulation. The predominant use of general anesthesia in our cohort, rather than regional techniques, may also influence thermoregulation through different effects on peripheral vasodilation and metabolic rate. In the post-operative phase, typically within the purview of intensive care or post-anesthesia care units, continued monitoring for rebound hypothermia or hyperthermia and management of shivering are important considerations. Future studies should account for these surgical variables through stratified analysis or randomized designs.

This study has several limitations. First, the sample size of 120 patients is relatively small, which may limit the statistical power and increase the risk of overfitting, especially given the number of variables included in the LASSO and logistic regression analyses. Although internal validation using Bootstrap resampling was performed, the model’s generalizability remains uncertain. Second, the model was validated only internally; external validation using an independent, multi-center cohort is necessary to confirm its robustness and clinical applicability. Third, this study focused solely on intraoperative hypothermia and did not assess long-term outcomes such as postoperative infections or functional recovery. Future studies should include larger, prospective cohorts and external validation to enhance the model’s reliability and utility in clinical practice.

## 5 Conclusion

In this retrospective cohort study, intraoperative fluid volume, operating room temperature, anesthesia duration, and ALB were identified as risk factors for IHC in elderly joint replacement patients. The risk prediction nomogram model constructed based on these factors demonstrated predictive value for the risk of IHC. The methodology employed, including LASSO regression for variable selection and logistic regression for model building, along with internal validation, supports the robustness of these findings in addressing the study’s primary aim. Clinically identifying high-risk populations early and implementing targeted nursing strategies holds significant value for improving patient outcomes.

## Data Availability

The original contributions presented in the study are included in the article/supplementary material, further inquiries can be directed to the corresponding authors.
